# Implementing landscape genetics in molecular epidemiology to determine drivers of vector-borne disease: A malaria case study

**DOI:** 10.1111/mec.16846

**Published:** 2023-02-01

**Authors:** Alfred Hubbard, Elizabeth Hemming-Schroeder, Maxwell Gesuge Machani, Yaw Afrane, Guiyun Yan, Eugenia Lo, Daniel Janies

**Affiliations:** 1 Department of Bioinformatics and Genomics, University of North Carolina at Charlotte, North Carolina, Charlotte, USA; 2 Center for Computational Intelligence to Predict Health and Environmental Risks (CIPHER), University of North Carolina at Charlotte, Charlotte, North Carolina, USA; 3 Department of Microbiology, Center for Vector-borne Infectious Diseases (CVID), Colorado State University, Fort Collins, Colorado, USA; 4 Centre for Global Health Research, Kenya Medical Research Institute, Kisumu, Kenya; 5 Department of Medical Microbiology, University of Ghana Medical School, Accra, Ghana; 6 Program in Public Health, University of California, Irvine, California, USA; 7 Department of Biological Sciences, University of North Carolina at Charlotte, Charlotte, North Carolina, USA; 8 School of Data Science, University of North Carolina at Charlotte, Charlotte, North Carolina, USA

**Keywords:** landscape genetics, molecular epidemiology, *Plasmodium falciparum*, relatedness, resistance surface, vector-borne disease

## Abstract

This study employs landscape genetics to investigate the environmental drivers of a deadly vector-borne disease, malaria caused by *Plasmodium falciparum*, in a more spatially comprehensive manner than any previous work. With 1804 samples from 44 sites collected in western Kenya in 2012 and 2013, we performed resistance surface analysis to show that Lake Victoria acts as a barrier to transmission between areas north and south of the Winam Gulf. In addition, Mantel correlograms clearly showed significant correlations between genetic and geographic distance over short distances (less than 70 km). In both cases, we used an identity-by-state measure of relatedness tailored to find highly related individual parasites in order to focus on recent gene flow that is more relevant to disease transmission. To supplement these results, we performed conventional population genetics analyses, including Bayesian clustering methods and spatial ordination techniques. These analyses revealed some differentiation on the basis of geography and elevation and a cluster of genetic similarity in the lowlands north of the Winam Gulf of Lake Victoria. Taken as a whole, these results indicate low overall genetic differentiation in the Lake Victoria region, but with some separation of parasite populations north and south of the Winam Gulf that is explained by the presence of the lake as a geographic barrier to gene flow. We recommend similar landscape genetics analyses in future molecular epidemiology studies of vector-borne diseases to extend and contextualize the results of traditional population genetics.

## 1 | BACKGROUND

Progress towards malaria elimination has stalled (WHO, 2021), in part because an inadequate understanding of how the environment influences transmission has hampered epidemiological modelling and targeting of control measures (Rabinovich et al., 2017). The *Anopheles* mosquitoes that transmit malaria rely on favourable environmental conditions to feed and reproduce successfully and human movement patterns that spread malaria are influenced by the available infrastructure. This means the environment plays a critical role in understanding and combating malaria transmission (Castro, 2017).

Several prior studies have sought to explain the environmental drivers of malaria transmission using methods such as geographically weighted regression and Bayesian risk modelling (Canelas et al., 2016). The former is logical for demographic or socioeconomic drivers that are tied to the host, but it does not capture the full variability in environmental drivers. Malaria transmission and gene flow can occur across a wide range of geographic scales, and thus, models that include the space between sample locations will yield greater insights. Risk maps inferred with Bayesian methods or kriging can be compared with environmental data layers for a more spatially distributed understanding of the drivers of transmission, but these methods typically do not incorporate varying levels of connectivity and gene flow between different locations.

In previous studies, we have addressed these limitations using resistance surface models that seek to explain genetic distances between populations in terms of environmental or resistance distances (Kepple et al., 2021; Lo, Hemming-Schroeder, et al., 2017; Lo, Lam, et al., 2017). These distances represent the difficulty of travelling between two locations in a manner that considers both geographic distance and the properties of the intervening landscape, allowing assessment of which landscape properties obstruct or enable gene flow. Although parasites do not directly traverse the landscape, the landscape indirectly influences parasite gene flow through the impact of the environment on the movements of parasite hosts (i.e., mosquitoes and humans). Thus, resistance surfaces are a promising tool for studying vector-borne disease (Hemming-Schroeder et al., 2018). Our prior work has begun to build a better understanding of the spatial determinants of malaria transmission, but small numbers of study sites have limited the scope of the conclusions.

In this study, we use resistance surface analysis to examine the drivers of malaria transmission in Western Kenya, a malaria endemic area with moderate-to-high levels of transmission. This is the first time these methods have been applied in malaria using more than 10 study sites. We also perform conventional population genetics analyses, such as Bayesian- and ordination-based clustering techniques, to contextualize our landscape genetics results and enable comparison with the existing body of literature. We show novel patterns and drivers of genetic differentiation, and thus, heterogeneity in transmission, and provide a rigorous demonstration of the utility of landscape genetics in the study of vector-borne diseases.

## 2 | MATERIALS AND METHODS

### 2.1 | Scientific and ethical statement

Scientific and ethical clearance for sample collection and preparation was given by the institutional scientific and ethical review boards of the Kenya Medical Research Institute, Kenya, and the University of California, Irvine, USA. Written informed consent/assent for study participation was obtained from all consenting heads of households, parents/guardians (for minors under the age of 18), and each individual who was willing to participate in the study.

### 2.2 | Study area

Malaria transmission in Western Kenya is moderate to high with contemporaneous prevalence estimates ranging from 40% to 60% in the lowlands near Lake Victoria (Okoyo et al., 2015; Zhou et al., 2016) to around 15% in the highlands (Zhou et al., 2016). There are pronounced gradients in elevation, rainfall and temperature in our study region (Figure S1). Temperature and moisture are both crucial to mosquito survival and activity, and the reduced, seasonal malaria transmission observed in the highlands of Kenya is explained by lower temperature, rainfall and humidity (Kenya NMCP, KNBS, & ICF International, 2016).

### 2.3 | Sample collection and genotyping

**FIGURE 1:**
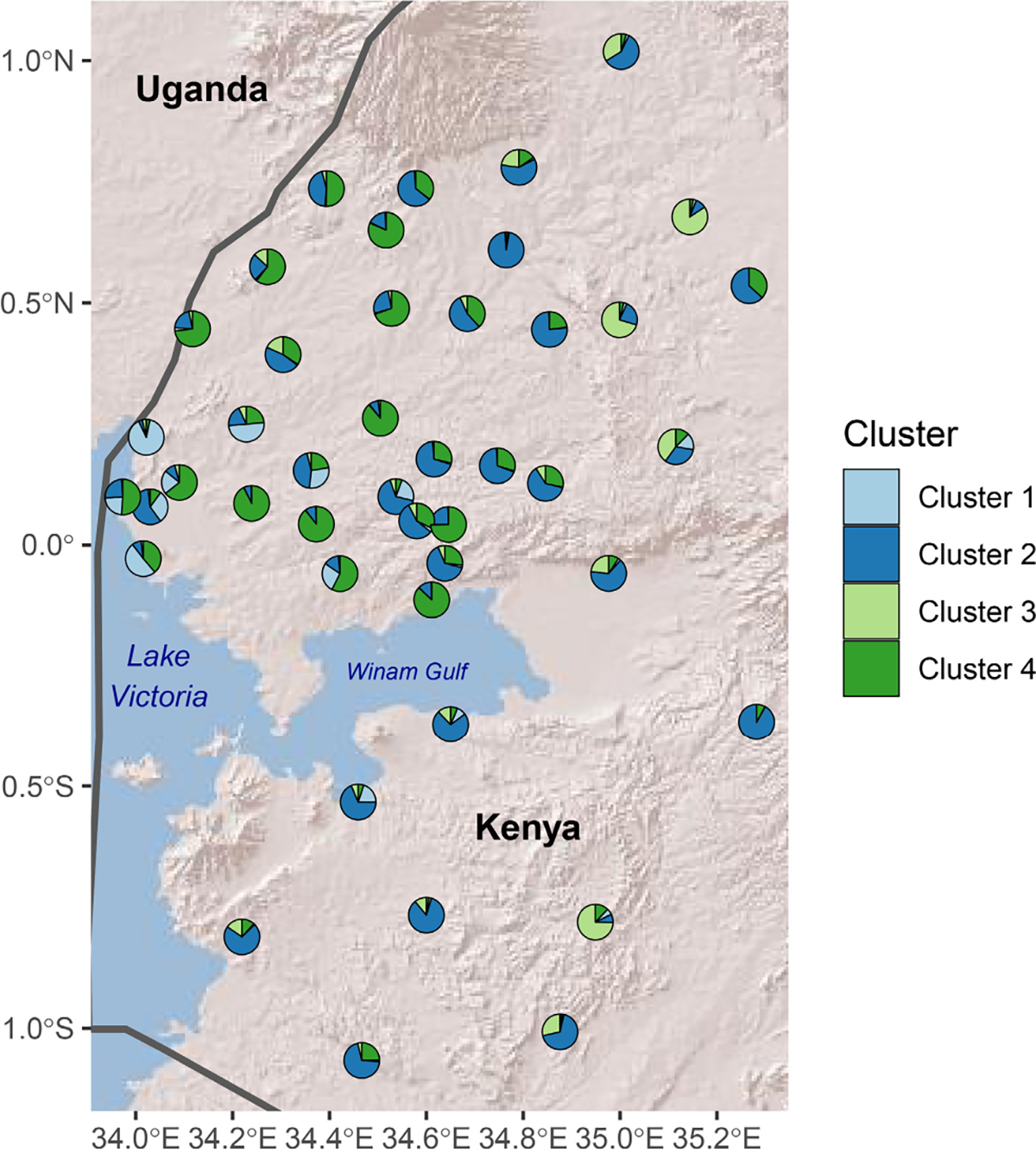
Map with pie charts showing average admixture coefficients (i.e., proportion membership in each cluster) for the samples from each study location, as estimated by *rmaverick*. The scatterpie R package (Yu, 2021) was used to create the pie charts. Country boundaries are included for context, obtained with the rnaturalearth R package (South, 2017). The background is Esri’s World Shaded Relief layer (^©^ 2009 ESRI).

A total of 1804 PCR-confirmed *P. falciparum* DNA samples collected in 2012 and 2013 across 44 sites in Western Kenya (Figure 1) were included. These samples were selected from 11,000 asymptomatic school children aged 3–12 years, as described in our earlier study (Lo et al., 2015). Approximately 50 μl of blood collected by finger prick was blotted on Whatman 3MM filter paper, from which *P. falciparum* DNA was extracted using the Saponin/Chelex method (Bereczky et al., 2005).

Eight single-copy microsatellite loci (Table S1) were genotyped for *P. falciparum*. Each PCR involved 2 μl of genomic DNA in 2 mM MgCl^2^, 2 μM of each primer (forward primers were labelled with fluorescent dyes; Applied Biosystems) and 10 μl of 2×DreamTaq Green PCR Master Mix (Thermo Scientific). PCR cycling conditions were as follows: 2 min, 94 C; (30 s, 94 C; 40 s, 58 C; 50 s, 72 C) for 40 cycles; and 5 min, 72 C. After amplification, the products were combined into three groups based on size and separated on an ABI 3730 sequencer. The allele sizes were recorded using two methods, depending on the sample: manual visualization using Peak Scanner and automated extraction in the Thermo Scientific Cloud Microsatellite Analysis Software. In both cases, a threshold of 300 relative fluorescent units was used for peak detection to filter out background noise. For each microsatellite, the dominant allele and any other alleles with at least 33% of the dominant allele’s height were scored. Five hundred and seventy-four samples were processed with both methods, and 79% of the overlapping alleles were scored identically. The automated method was considered more accurate and was used for alleles scored with both methods.

Samples were filtered by both the number of successfully scored loci per sample and the number of samples per study site. Only samples with at least six successfully scored loci were included in further analyses. This ensured that every pair of samples would have at least four overlapping loci. The *P. falciparum* samples were grouped into populations according to the clinic where they were collected (i.e., the geographic location). Of these populations, only those with at least five samples were used in further analyses. 81.3% of samples passed both filters. The preprocessed microsatellite data are provided in Table S2 and the study site locations are provided in Table S3.

### 2.4 | Population structure

Linkage disequilibrium (LD) was estimated by computing the ${\overset{‾}{r}}_{d}$ statistic, which approximates the popular index of association but does not increase with the number of loci (Agapow & Burt, 2001). This was computed with the poppr R package (Kamvar et al., 2014), both with and without clonal correction. Pairwise LD was also estimated for each unique pair of loci. Missing data values were ignored for these computations.

Genetic clustering of samples was first assessed with principal component analysis (PCA), a method which transforms the input matrix into a set of orthogonal components ranked in descending order of the variance they explain. Visualizing combinations of the highly ranked components allows one to identify the number of clusters present in the data. In this case, the microsatellite genotypes were converted into binary format, meaning one column per locus-allele combination, one row per sample, and a value of 0 or 1 based on whether that allele was present. This format was chosen because it allows flexible representation of samples with multiple clones. Missing data values were replaced with the mean frequency of the allele in question, as this allows all samples to be used in PCA without biasing the results. PCA was then performed on this binary matrix using the R programming language (R Core Team, 2021) and visualized with the aid of the GGally R package (Schloerke et al., 2021). To corroborate the PCA findings, another ordination technique, discriminant analysis of principal components (DAPC), performed with the adegenet R package (Jombart, 2008), was also used to estimate the number of clusters (Appendix S1).

Principal component analysis shows the overall pattern of clustering in a genetic data set, but PCA cannot be used to estimate the degree of membership a given individual has in a given population. In addition, genetic data will likely not conform to the linearity assumption of PCA. For this reason, PCA was only used to identify the probable number of clusters present in the data. Another programme, *rmaverick*, was used to assign individuals to clusters and estimate admixture coefficients for each individual (Verity, 2018). *rmaverick* is a Bayesian method that, similar to the popular programme STRUCTURE (Pritchard et al., 2000), seeks to find the population groups that are not in Hardy–Weinberg or linkage equilibrium. The admixture coefficients for each individual represent the proportion of membership that individual has in each cluster. Unlike alternatives like STRUCTURE, *rmaverick* uses a Metropolis-coupled Markov chain Monte Carlo technique, in which parameter values are simultaneously estimated in different ‘chains’ and information is periodically passed between chains (Verity & Nichols, 2016). This information passing improves mixing and can make it more likely that the model will converge.

Based on evaluation of different parameter configurations, *rmaverick* was run with 10,000 burn-in iterations, 2000 sampling iterations, 500 rungs, a GTI power of 3 and the admixture model. The burn-in iterations are an initial period in which the model is run without saving results to avoid bias from the initial conditions, whereas the sampling iterations are the portion of the model run in which results are saved. The rungs are the number of Metropolis-coupled chains used and the GTI power controls the distribution of these chains. Mono- and biclonal infections were incorporated by running *rmaverick* with mixed ploidy and repeating the allele when only one was present for a given locus. Samples with more than two clones were discarded for this analysis. The pophelper R package (Francis, 2016) was used to assist in visualization of results.

### 2.5 | Spatial patterns of relatedness

Genetic relatedness between samples was estimated as the proportion of shared alleles, treating each polyclonal infection as a single ‘subpopulation’, given that no existing analytical method can separate individual parasite haplotypes for infections that have multiple alleles at more than one locus. Each infection was treated as a subpopulation when calculating genetic relatedness, similar to the approach of Wesolowski et al. (2018). Thus, the value we obtained was the proportion of shared alleles between samples or infections, but not necessarily between individual parasite clones. The specific algorithm used to compute the proportion of shared alleles was that employed in the R package PopGenReport (Adamack & Gruber, 2014), reimplemented to work with our data. Missing reads were treated as the absence of any alleles for the locus in question and did not affect the calculation.

This individual-based measure was then aggregated to the population level by taking the fraction of individual relationships that passed a relatedness threshold of 0.15 and converted to genetic distance by subtracting from 1. In other words, the relatedness for all of the pairs of individuals corresponding to each pair of populations was compared with this threshold and the population-based measure of relatedness was taken to be the fraction of individual pairs that exceed the threshold. This fraction of ‘highly-related’ individuals should be more sensitive to recent demographic events than other metrics such as average relatedness (Taylor et al., 2017). After sensitivity testing using thresholds between 0.1 and 0.3, we selected a threshold of 0.15 because this did not lead to saturation at either the lower or upper bounds (i.e., clumping of pairs near a genetic distance of 0 or 1; Figure S2). This threshold is considerably lower than that used by Taylor et al. (2017), but they used single-nucleotide polymorphisms, rather than microsatellite data. The relatedness expected by chance alone is much higher in the former case. The distribution of the final relatedness values is shown in Figure S3.

To identify clustering of genetic information in geographic space, an ordination technique named MEMGENE was used (Galpern et al., 2014). MEMGENE extends PCA to isolate the spatial portion of the variance in a matrix of genetic distances. MEMGENE does this by performing PCoA on the matrix of geographic distances to find components that represent the geographic patterns among study sites, regressing these components against the genetic distances and then performing a second PCoA on the regression predictions. The components of this second PCoA are the MEMGENE variables, and each one can be thought of similarly to the components from standard PCA, except they only represent the portion of genetic variance that can be explained by geographic patterns. Visualizing these MEMGENE variables at each study location can show spatial clusters of related populations and point to possible barriers to transmission. This analysis was performed with the matrix of population-level genetic distances described previously.

To evaluate the hypothesis of isolation-by-distance (IBD), the study site coordinates were reprojected into planar space and geographic and genetic distances were compared using both a Mantel correlogram and the test for congruence among distance matrices (CADM). Coordinate reprojection converts the elliptical coordinate space that describes the Earth’s curved surface into a two-dimensional space that is amenable to analysis. The map projection selected for this study was the WGS 84-based coordinate system for UTM zone 36 N. Once reprojected, Euclidean distance was calculated between all pairs of study sites. Mantel correlograms (Oden & Sokal, 1986) were computed using the R package vegan (Oksanen et al., 2020) using these Euclidean distances and the genetic distances described previously. The R package ape (Paradis & Schliep, 2019) was used to test for CADM.

### 2.6 | Landscape genetics

Environmental variables that either have been previously associated with *P. falciparum* transmission or that influence host and/or vector movement were selected for inclusion in resistance surface analysis (Table 1). To evaluate a potential barrier effect from Lake Victoria, a binary layer was created that represented grid cells belonging to Lake Victoria with a 1 and other grid cells with a 0. The rainfall and LST data, which are available at subannual frequency, were aggregated for the entire year using a mean composite. Both 2012 and 2013 were used for these aggregations, but collection of the LST data did not begin until 25 January 2012, so the first part of January 2012 is not included. The land cover data set was created at an annual time scale, and the 2012 version was selected for this study. The DEM and friction to human movement data sets are static, and, therefore, these considerations do not apply. All data sets were reprojected into the WGS 84-based UTM 36 N coordinate system with 1 km spatial resolution.

Using these environmental data sets and the matrix of population-based genetic distance, resistance surfaces were estimated to assess the degree to which each environmental variable explains the observed patterns of genetic relatedness. Resistance surfaces are gridded spatial layers in which the values of each cell represent the degree to which that space obstructs gene flow. By treating gene flow as a proxy for transmission, the surface as a whole represents areas that are more or less permissible to malaria transmission. We used the R package ResistanceGA (Peterman, 2018) to optimize resistance surfaces. Briefly, this process involves (1) finding the least cost path between every pair of locations through the current resistance surface; (2) fitting a mixed linear effects model that explains genetic distance in terms of this resistance distance; and (3) applying a transformation to the resistance surface to improve the fit. This process is iterated many times. In the first round, the resistance surfaces are simply the rescaled environmental inputs. The entire procedure is performed in the framework of a genetic algorithm that tests a certain number of mutations (transformations) per generation, chooses the most fit to carry to the next generation (based on the mixed linear effects model) and repeats until the change in fitness does not meet a certain threshold.

For each run, ResistanceGA merges the input layers into a single composite layer, which serves to incorporate multiple inputs without creating multicollinearity issues. ResistanceGA accomplishes this compositing by summing the surfaces after transformations have been applied, which ensures this operation is mathematically rational, even for categorical variables. All possible combinations of input layers, including each layer individually, were tested. Because multiple input layers are transformed into one variable prior to fitting the regression models, the multicollinearity issues in landscape genetics described by Prunier et al. (2015) are not a concern.

Each resistance surface was fit twice so that consistency between replicates could be assessed. After fitting the resistance surfaces, bootstrapping was performed to determine how robust the fit of each surface is to random subsets of the input samples.

### 2.7 | Software pipeline

Unless otherwise noted, all analyses were performed using custom code written in the R (R Core Team, 2021) and Python (Python Software Foundation, 2022) programming languages. Throughout, the adegenet R package (Jombart, 2008) was used for reformatting genetic data, the Geospatial Data Abstraction Library (GDAL/OGR contributors, 2021) and the raster (Hijmans, 2022) and sf (Pebesma, 2018) R packages were used for spatial data processing, and the R tidyverse packages (Wickham et al., 2019) were used for general data manipulation and visualization. knitr (Xie, 2014) and R Markdown (Xie et al., 2018, 2020) were used to organize and document analyses. The entire workflow was automated with Snakemake (Mölder et al., 2021) and is available on Bitbucket at https://bitbucket.org/a-hubbard/hubbardetal_landgen_drivers_malaria/. This repository includes specifications of the exact versions of each package used.

## 3 | RESULTS

### 3.1 | Population structure

Linkage disequilibrium analysis shows weakly significant LD driven by a single pair of loci (TA42 and 9735). This is consistent with weak population structure in a high transmission environment.

The PCA results suggested that the potential number of distinct genetic clusters is between three and six. The first four components of the PCA explain 5.9%, 3.8%, 3.6% and 3.2% of the overall variance, respectively. Visualization of these components shows two to three distinct clusters delineated by the first component and two weakly separated clusters in the third component (Figure S4). The other components explained less than 3% of the overall variance and thus were not analysed in detail. DAPC indicated three or four genetic clusters (Appendix S1), providing further evidence that somewhere between three and six clusters are supported by the data.

Based on these PCA and DAPC results, *rmaverick* was run for *K* values from one to six. The admixture bar plots show considerable mixing overall but some structuring according to latitude (Figure S5a) and elevation (Figure S5b). To visualize these geographic patterns in more detail, pie charts depicting mean population admixture coefficients were visualized at each study location for a *K* of four (Figure 1). Although there is no ‘true’ *K*, the model with four clusters was best supported by the posterior evidence (Figure S6) and so was a logical choice for further inspection. Inspection of Figure 1 indicates some differentiation on the basis of geography and elevation. Samples with substantial membership in cluster 1 primarily came from the western portion of the study region, near the border with Uganda. Cluster 4 has some overlap with this area, but encompasses a wider area covering all of the lowlands north of the Winam Gulf. Cluster 3 is primarily associated with samples from higher elevation sites in the eastern portion of the study area. Cluster 2 does not seem to be strongly tied to geographic factors. To corroborate these findings and provide a reference for readers more familiar with STRUCTURE, we performed analogous investigations with this software, which yielded similar results (Appendix S2).

### 3.2 | Spatial patterns of relatedness

**FIGURE 2:**
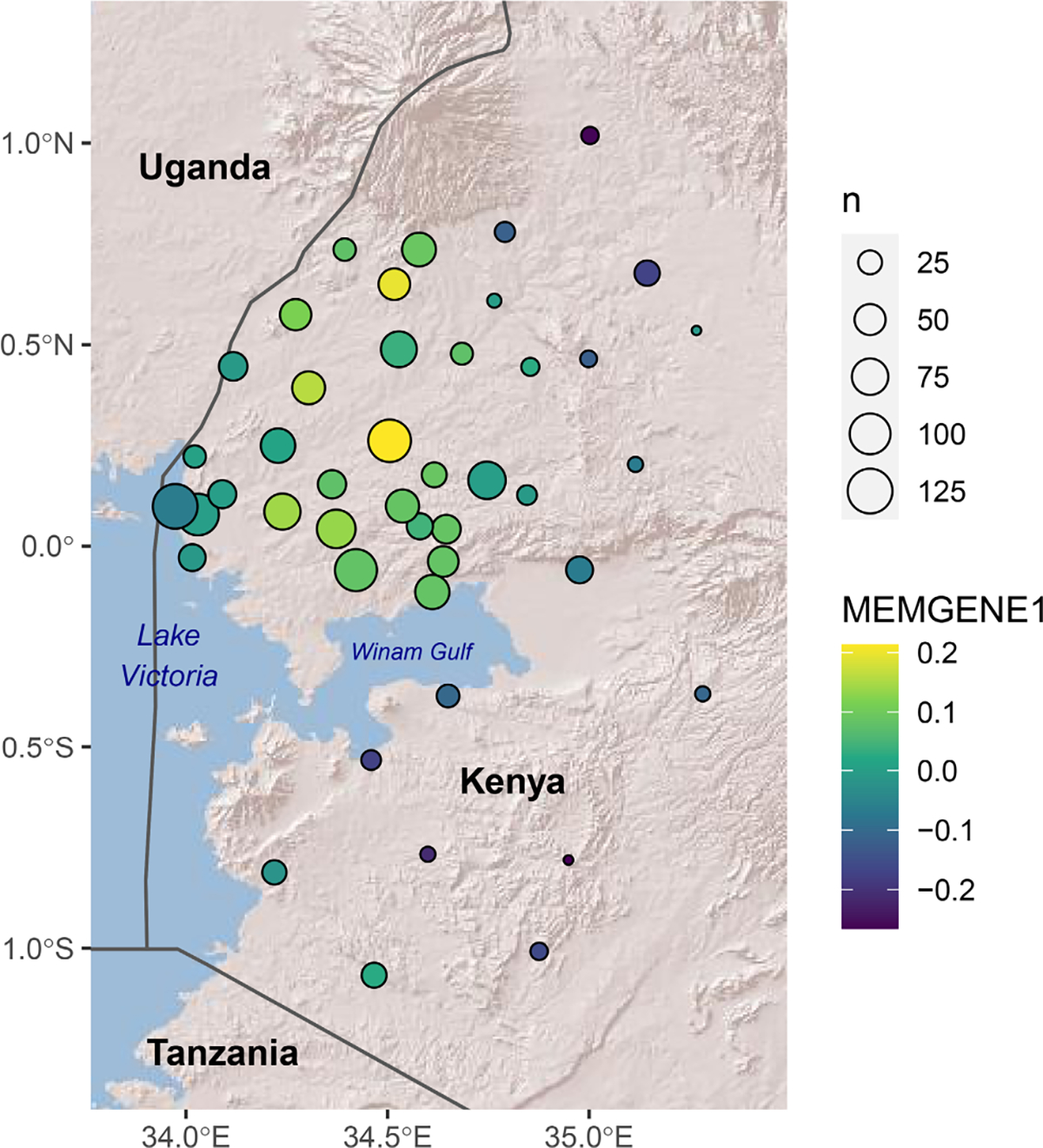
Map showing sample site locations with the point colour scaled according to the first MEMGENE variable and the size representing the number of samples gathered at that location. Country boundaries are included for context, obtained with the rnaturalearth R package (South, 2017). The background is Esri’s World Shaded Relief layer (^©^ 2009 ESRI).

MEMGENE showed distinct spatial clusters north and south of Lake Victoria. This analysis revealed that 8.3% of the overall variance in genetic distances can be explained by spatial patterns. Of this fraction, the first component, or MEMGENE variable, explained 45.7% and showed a distinct spatial cluster of genetic similarity in the lowlands north of the Winam Gulf of Lake Victoria (Figure 2). The areas south and east of the Winam Gulf comprise a second cluster. Samples gathered near the Ugandan border, in the northwest of the study area, fall somewhere in between, but bear more similarity to samples from the south and east. The other components explained a considerably lower fraction of the spatial portion of the variance, and thus, a very low fraction of the overall variance, and so were not visualized. After the regression step in MEMGENE (see Materials and Methods), a redundancy analysis is performed to identify components that significantly improve fit. The results described previously were found with a significance threshold of 0.05 in this step. When this threshold was lowered to 0.01, no significant components were found, suggesting the pattern described previously is only weakly significant.

**FIGURE 3:**
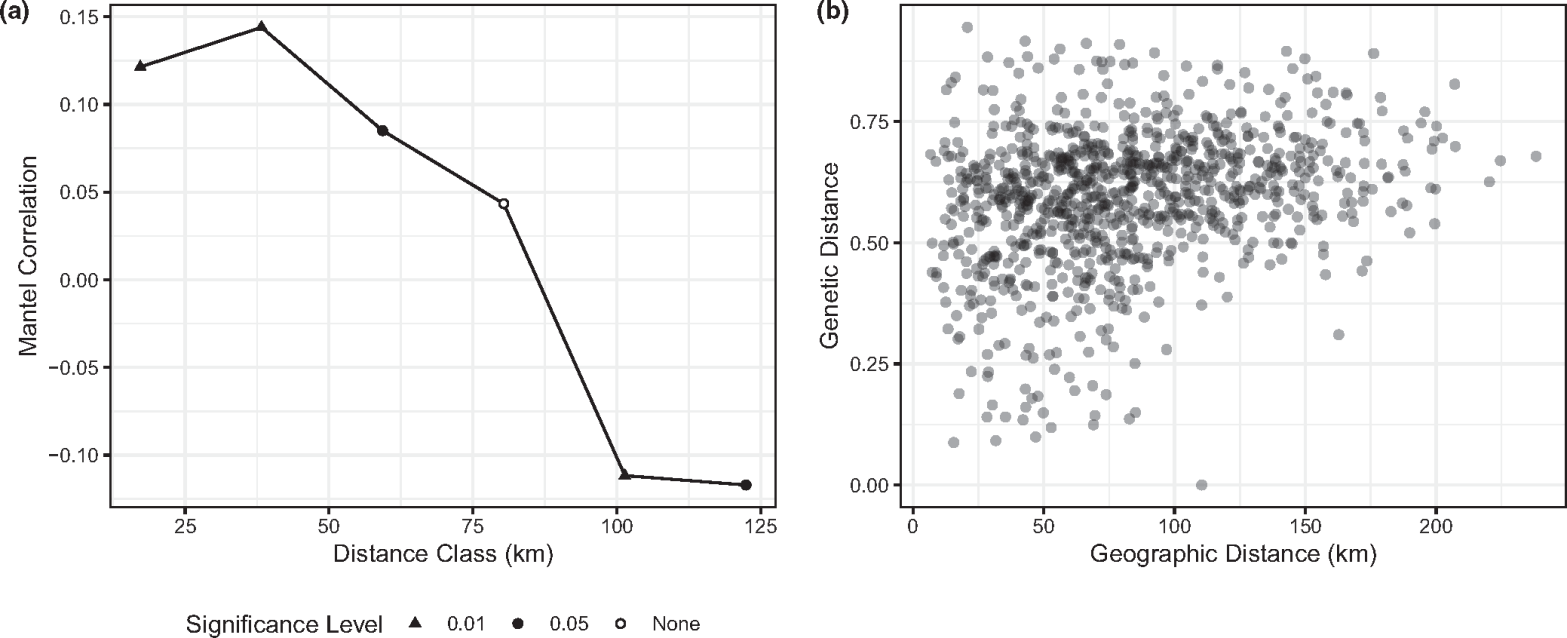
Mantel correlogram showing the correlation between genetic and geographic distance (a). In this type of plot, the x-axis is a series of geographic distance classes and the y-axis is the correlation between genetic and geographic distance in samples separated by this distance class. Point shape indicates the significance level of each correlation. For reference, the distance-distance plot of geographic versus genetic distance is also included (b). Note that only distance classes with adequate numbers of samples for analysis were included in the Mantel correlogram, which is why the x-axes do not have the same extent.

The Mantel correlogram shows significant correlation between genetic and geographic distance over short distances (*p* < 0.05 for less than 70 km; Figure 3a, Table S4). This pattern of IBD was corroborated by the test for CADM, which showed highly significant congruence between genetic and geographic distance matrices (*W* = 0.642; *p* = .00045). However, the correlogram shows this relationship disappears as geographic distance increases, until eventually significant negative correlation was found at higher geographic distances. This surprising result can be understood by studying the MEMGENE map discussed above. While the majority of the sites in the second cluster, corresponding to negative MEMGENE values, are south and east of the Winam Gulf, several of the sites near the Ugandan border north of the Gulf also belong to this cluster (Figure 2). Many of these sites are between 90 and 130 km from the other locations belonging to this cluster, south and east of the Gulf, which correspond to the distances where negative correlations are observed in the Mantel correlogram. This suggests that a process that is not well-represented by geographic distance alone is driving genetic similarity between these two locations.

### 3.3 | Landscape genetics

The resistance surfaces clearly show that Lake Victoria is acting as a barrier to gene flow, based on both the ranking of best-fitting surfaces and high resistance values over Lake Victoria. To rank the surfaces, the corrected Akaike information criterion (AICc) was used with all output surfaces generated by both replicates. This is displayed for the 10 best-fitting surfaces in Table 2, along with the number of parameters and the conditional and marginal *R*^2^. Generally speaking, the two replicates conducted for each set of inputs did not produce identical outputs. However, the differences in likelihood and AICc were always small (Table S5), suggesting similar solutions and goodness-of-fit had been obtained between replicates. For the sake of comparing variables, the surface from the best-fitting replicate was selected for each set of inputs for display in Table 2 and visual inspection. Most of the best-fitting surfaces contained the binary Lake Victoria layer. LST and friction to human movement without access to motorized transport were also consistently present in the top-ranking surfaces. The distance-only and null models did not rank particularly highly, indicating the best landscape resistance models explain patterns of gene flow that geographic distance alone cannot. All of these conclusions were corroborated by the bootstrapping results (Table S6).

**FIGURE 4:**
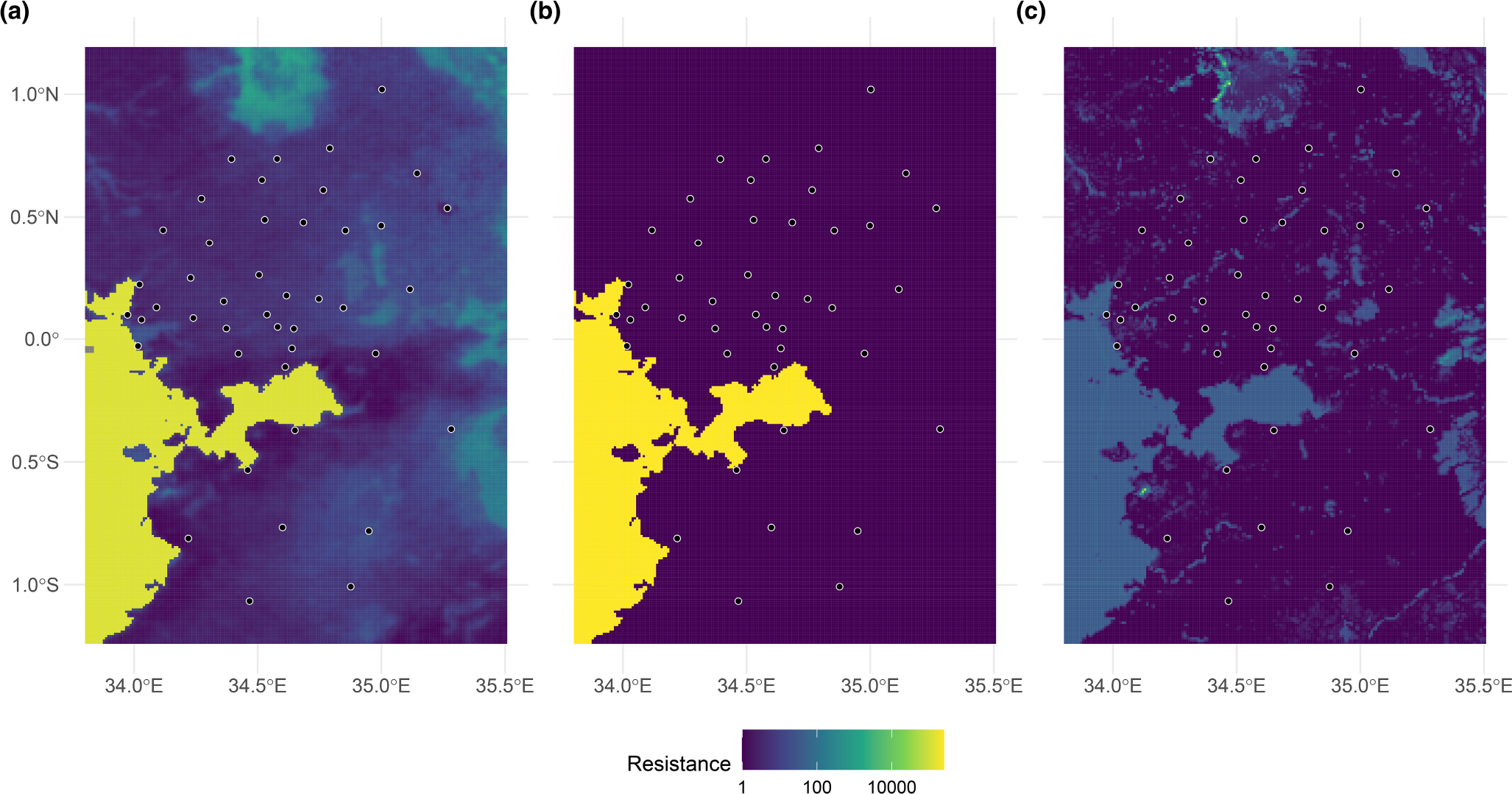
The three best-fitting resistance surfaces, ranked in order of fit: a composite surface of LST and the Lake Victoria binary layer (a), a single surface modelled off the Lake Victoria binary layer (b), and a single surface modelled off the friction to human movement data set that does not assume access to motorized transport (c). Resistance values are log_10_ transformed, and study site locations are shown for context. The surfaces were visualized with the landscapetools (Sciaini et al., 2018) and patchwork (Pedersen, 2020) R packages.

In the resistance surfaces themselves (Figure 4), pixels associated with Lake Victoria were assigned high resistance values in all of the best-fitting surfaces, regardless of whether they included the binary Lake Victoria layer. However, in the highest ranked layer, Lake Victoria and LST, LST was weighted to contribute more to the final model (77%), indicating that variable explains a substantial amount of variance that Lake Victoria alone cannot. Low land surface temperature was associated with high resistance to gene flow, as was high friction to human movement without access to motorized transport. Of the other environmental covariates, high resistance to gene flow was associated with high elevation, high friction to human movement with access to motorized transport, low precipitation and water bodies in the land cover layer (results not shown).

## 4 | DISCUSSION

The results presented in this study support an isolation-by-barrier (IBB) hypothesis, where Lake Victoria acts as an obstacle to gene flow between the northern and southern parts of our study area. The *rmaverick* analysis gave the first indication of this conclusion, in that samples collected from north and south of the Winam Gulf of Lake Victoria tended to have membership in different genetic clusters. The pattern of spatial clustering in the first MEMGENE variable showed the same result, with one geographic cluster of genetic similarity in the lowlands north of Lake Victoria and the other encompassing the areas east and south of the lake. Finally, the resistance surface analysis suggested both that Lake Victoria was an important variable in dictating landscape resistance, as seen through the ranking of best-fitting surfaces, and that the lake is associated with a high resistance to gene flow, as evidenced by the surfaces themselves.

Other studies using data from a similar time period and region have by- and-large shown high gene flow (Nderu et al., 2019) leading to little genetic differentiation among parasite populations (Ingasia et al., 2016; Nderu et al., 2019; Nelson et al., 2019), although in one case this varied somewhat based on the genetic distance measure used (Nelson et al., 2019). A study conducted with more recent data (dating to 2018 and 2019) supported the same conclusion of little differentiation between populations (Onyango et al., 2021). Our results are qualitatively consistent with these findings, but cannot be compared quantitatively as these studies used different measures of genetic distance.

Our results are also consistent with previous investigations into the clustering of genetic relatedness in this area. Ingasia et al. (2016) showed with PCA that samples from Kisii, located in the highlands south of the Winam Gulf, clustered separately from samples collected from the lowlands north of the Gulf (Kisumu and Kombewa) and the highlands east of the gulf (Kericho). Omedo, Mogeni, Rockett, et al. (2017), using spatial scan statistics, identified a cluster of genetic similarity in part of the area north of the Winam Gulf, near the border with Uganda. Our findings indicate a distinct population north of the Winam Gulf, as well as some evidence of differentiation between highlands and lowlands. However, we also found a handful of sites near the Ugandan border that did not cluster with the rest of the sites north of the Gulf, but were more similar to samples collected south and east of Lake Victoria. These sites may correspond to the cluster found by Omedo, Mogeni, Bousema, et al. (2017) and are separated from the rest of Kenya by the Nzoia River, possibly explaining why they are distinct from the remainder of the lowlands north of the Gulf. The similarity with sites to the south and east of the Lake is less intuitive but may be explained by patterns of long-distance human movement, which Wesolowski et al. (2012) found to be common in the Lake Victoria region. Another study in this area, Omedo, Mogeni, Bousema, et al. (2017), did not find any clustering from PCA, but they were focussed on a small subset of our study region (Rachuonyo South).

Previous studies investigating isolation-by-distance in western Kenya have yielded inconsistent results. Qualitative assessments of IBD have found none (Ingasia et al., 2016; Nelson et al., 2019), but more formal tests have shown some significant correlations between genetic and geographic distance at or below distances of 20 km between sites (Omedo, Mogeni, Bousema, et al., 2017; Omedo, Mogeni, Rockett, et al., 2017). This has some similarity to our findings, in that we discovered weakly significant correlations between geographic and genetic distance in distance classes at or below 60 km. Taken together, this suggests that some IBD has occurred in *P. falciparum* populations in this region, but it is only noticeable at relatively short distances (0–60 km, depending on the study and methods) and between certain locations.

In terms of IBB and IBR, few studies have been performed on malaria, but those that do exist for our study region did not identify Lake Victoria as a barrier to gene flow. Ingasia et al. (2016) informally described isolation between highland and lowland populations, while Omedo, Mogeni, Rockett, et al. (2017) looked for a barrier more formally and found nothing. The first result is not inconsistent with our own. Ingasia et al. (2016) had relatively few study sites, with only one each north and south of the Winam Gulf. They may not have had the spatial coverage to identify a barrier effect from Lake Victoria, and their finding of isolation between highlands and lowlands is supported to some extent by our own clustering analyses. The contrasting conclusions on the presence of a barrier in our study and Omedo, Mogeni, Rockett, et al. (2017) may also come down to methodological differences. That study used a regression framework in which each 10×10 km pixel in the study region was treated as a separate variable and barrier effects were assessed for pixels separating site pairs where the straight line connecting the two sites passed through the pixel in question. By contrast, ResistanceGA fits values of high or low resistance to environmental covariates in their entirety (Peterman, 2018), rather than fitting different values in different parts of the study region. This makes our approach more suited to assessing the effect of environmental features holistically, throughout the study region, whereas the Omedo, Mogeni, Rockett, et al. (2017) method would be better suited to identifying barriers associated with small, specific geographic features similar in size to the 10×10 km pixels used in their model. In combination, then, these findings suggest that mixing is fairly homogeneous in the land areas of this study region, but that Lake Victoria, when considered as a single unit, does act as a barrier to gene flow between the northern and southern sides of the Winam Gulf.

Previous work on human movement in this area suggests relatively frequent travel within the Lake Victoria region (Blanford et al., 2015; Wesolowski et al., 2012). These studies did not clearly show Lake Victoria to be a barrier to movement, but they were intended for regional analyses and lacked the resolution to address this question in detail. One study, based on mobile phone data, does seem to indicate less connectivity with populations near the Ugandan border than in the rest of the Lake Victoria region (Wesolowski et al., 2012). This is consistent with the results of our clustering analyses and may explain the negative correlations revealed with the Mantel correlograms, but again, the resolution of that human movement study was such that we cannot be certain of this explanation.

In terms of vector populations, most research has considered Kenya as a whole and focussed on the differentiation between western and coastal Kenya (e.g. Ogola et al., 2019). Of the groups that studied western Kenya in particular, the results have been inconsistent. In one case, significant population structuring was found in *Anopheles gambiae s. l*. in the Lake Victoria region and, while other landscape factors were found to be more important, a landscape genetics analysis did show Lake Victoria to be an area of relatively low gene flow (Hemming-Schroeder et al., 2020). On the contrary, a more recent study done with the same species identified little genetic differentiation in this region and no apparent barrier in Lake Victoria, although no formal landscape genetics analysis was done (Onyango et al., 2022). However, only four study sites were used in this case, only one of which was south of the Winam Gulf, so it is likely this work lacked the spatial coverage to address these questions in detail. From this, we believe it likely that a large part of the barrier effect we discovered from Lake Victoria in *P. falciparum* genetics can be explained by the difficulty mosquitoes have traversing this large body of water.

Taken as a whole, these results indicate low overall genetic differentiation in the Lake Victoria region, but with some separation of populations north and south of the lake that is explained by the presence of the lake as a geographic barrier to gene flow. The resistance surface results suggest that both host and vector factors are important determinants of transmission, as friction to human movement and temperature, which will disproportionately affect mosquitoes, were both in the highest ranking surfaces.

This work is the most spatially comprehensive landscape genetics study done in malaria to date, and we have identified landscape impacts on gene flow, specifically a barrier effect from Lake Victoria, which have not been documented previously. However, this study does have certain limitations. First, while polygenomic microsatellites are relatively informative genetic markers, our study only used eight. Subsequent studies conducted with more genomic depth would be useful to confirm our findings. On a related subject, while our study has large sample sizes overall, some of the study locations only have a handful of samples, in particular south and east of Lake Victoria (Figure 2), which may bias our spatial analyses in those areas. In addition, the two separate genotyping methods used in our data set do represent a source of inconsistency. We have characterized the level of agreement between the two methods, but we did not attempt to repeat genotyping due to the age of the samples. Also, many of the environmental inputs used are proxies for the true variable of interest (e.g., LST as a proxy for near-surface air temperature). The data products selected are all well-correlated with those variables, but as more direct measures become available it will be important to repeat this and other landscape genetics analyses to confirm their findings. On the subject of spatial covariates, no information on spatial coverage of malaria control measures was included, despite the importance of these factors in driving population structure. There are no quality data on subnational spatial heterogeneity of coverage for most interventions, and for the one exception, insecticide-treated nets, little spatial heterogeneity was observed in our study area (Bertozzi-Villa et al., 2021). For this reason, these data were not included in our analyses. Finally, our conclusions rest on the assumption that the environmental data we used is reflective of the state of the landscape that is relevant to its impact on gene flow. In other words, we have implicitly assumed that the environment in 2012 and 2013, contemporaneous with sample collection, has the greatest impact on gene flow. In reality, the state of the environment prior to sample collection likely has had some impact. The relationship between contemporaneous and historical environmental variation and gene flow is a topic that deserves further research to characterize the lag time corresponding to the strongest correlation.

The results of this study have implications in a few different areas. For the sake of planning interventions, the populations around Lake Victoria are sufficiently connected that blanket control measures remain appropriate. However, it is probable that if interventions further reduce transmission in this area, these populations will become more distinct and interventions conducted in one area will have less impact on the other. In this situation, it would be recommended to consider populations north and south of the Winam Gulf as separate entities when targeting interventions.

For the modelling community, our results indicate that geographic distance is a poor proxy for transmission and that both vector and host factors can be important drivers of transmission at a moderate spatial scale. The first is an important finding because geographic distance is frequently used as a proxy for connectivity in models (Lee et al., 2021). More work is required to identify the best alternatives, but measures that represent heterogeneous patterns of transmission are necessary. In terms of the drivers of transmission, our study was performed at a reasonably coarse spatial scale, making it a surprise that an environmental variable that primarily impacts vector activity, LST, proved to be one of the most important explanatory variables. Further research is recommended to better understand how spatial scale impacts the drivers of transmission in vector-borne diseases, especially with respect to which scales are primarily governed by vector or host factors.

Finally, and most broadly, this study has demonstrated that landscape genetics analysis of vector-borne disease, when conducted with a large number of spatial locations, is capable of revealing and explaining barriers to gene flow in fairly high transmission settings that lack strong population structure. In future molecular epidemiology studies, we recommend that sensitive methods, such as MEMGENE (Galpern et al., 2014), first be used to characterize spatial heterogeneity in genetic variation. If significant variation is discovered, we recommend the use of landscape genetics methods, in particular resistance surface analysis, to explain the drivers of this structure. Doing so will extend and contextualize the results of traditional population genetics analyses and thus yield more insights into the spatial determinants of transmission.

## Supplementary Material

Supplemental figure S1-S6

Supplemental Text 1:Discriminant Analysis of Principal Components (DAPC)

Supplemental Text 2: STRUCTURE

Supplemental Table S1

upplemental Table S2

upplemental Table S3

upplemental Table S4

upplemental Table S5

upplemental Table S6

## Figures and Tables

**TABLE 1:** Spatial covariates included in resistance surface analysis.

Variable	Data set	Spatial resolution	Temporal resolution	Citation
Rainfall	Climate Hazards Group InfraRed Precipitation with Station (CHIRPS) data	0.05 degree	Daily	Funk et al. (2015)
Land surface temperature (LST)	MYD11A2.061	1 km	8 days	Wan et al. (2021)
Elevation	NASADEM	1 arc second	Static	NASA JPL. (2020)
Land cover	MCD12Q1	500 m	Annual	Friedl & Sulla-Menashe. (2019)
Lake Victoria binary layer	Derived from MCD12Q1	500 m	Annual	NA
Human mobility	Global human movement friction surface with access to motorized transport	1 km	Static (2019)	Weiss et al. (2020)
Human mobility	Global human movement friction surface without access to motorized transport	1 km	Static (2019)	Weiss et al. (2020)

**TABLE 2:** Fit statistics for top 10 best-fitting resistance surfaces.

Surface	*K*	*AIC_c_*	$R_{m}^{2}$	$R_{c}^{2}$
Lake Victoria and Land Surface Temperature	6	−1828.47	0.136461	0.495123
Lake Victoria	3	−1827.51	0.0674234	0.478118
Friction to Human Movement (w/o motorized)	4	−1826.23	0.0861454	0.48676
Elevation and Lake Victoria	6	−1823.91	0.130084	0.495179
Lake Victoria and Friction to Human Movement (w/o motorized)	6	−1822.71	0.0901474	0.491937
Land Surface Temperature	4	−1821.96	0.163029	0.515629
Land Surface Temperature and Friction to Human Movement (w/o motorized)	7	−1821.3	0.165838	0.518301
Lake Victoria and Precipitation	6	−1821.29	0.0501707	0.489653
Elevation and Friction to Human Movement (w/o motorized)	7	−1820.45	0.201408	0.53808
Lake Victoria and Friction to Human Movement (w/ motorized)	6	−1819.93	0.0672178	0.478748
