# Supplementary material for: Implementing landscape genetics in molecular epidemiology to determine drivers of vector-borne disease: A malaria case study
Source: Mol Ecol. Author manuscript; Available in PMC 2023 Dec 4. (PMC10694861; doi:10.1111/mec.16846)
Supplement: Supplemental figure S1-S6 [file NIHMS1941384-supplement-Supplemental_figure_S1-S6.pdf]

Implementing landscape genetics in molecular epidemiology to determine drivers of vector-borne disease: A malaria case study

Supplemental Figures

Alfred Hubbard Elizabeth Hemming-Schroeder Maxwell Machani
Yaw Afrane Guiyun Yan Eugenia Lo Daniel Janies

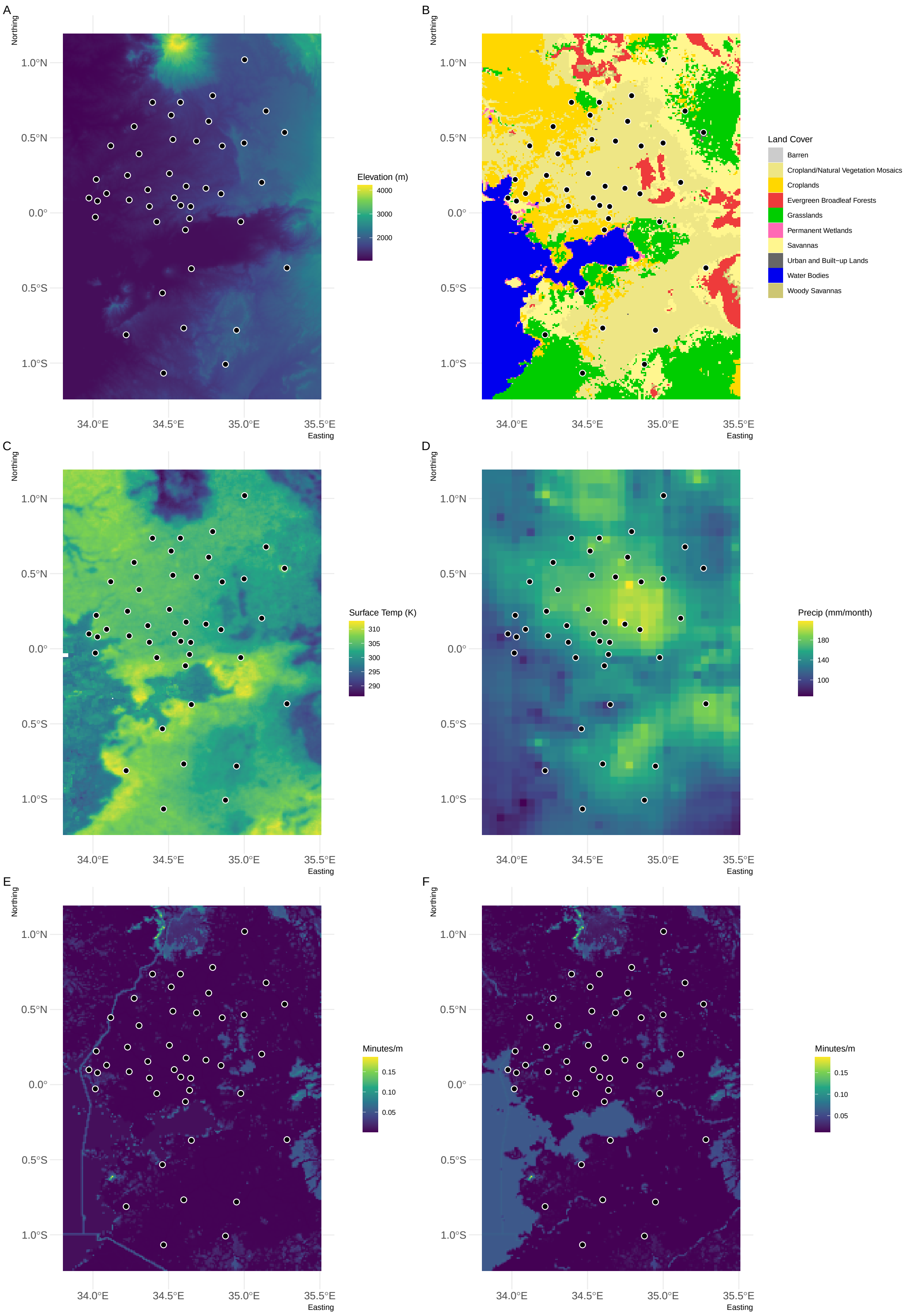
A
B
C
D
E
F
Northing
Easting
1.0°N
0.5°N
0.0°
0.5°S
1.0°S
34.0°E
34.5°E
35.0°E
35.5°E
Elevation (m)
4000
3000
2000
Land Cover
Barren
Cropland/Natural Vegetation Mosaics
Croplands
Evergreen Broadleaf Forests
Grasslands
Permanent Wetlands
Savannas
Urban and Built−up Lands
Water Bodies
Woody Savannas
Surface Temp (K)
310
305
300
295
290
Precip (mm/month)
180
140
100
Minutes/m
0.15
0.10
0.05

Supplemental Figure 1: Spatial covariates used in our study: elevation, from the NASADEM product **(A)**; land cover, from the MCD12Q1 product **(B)**; land surface temperature (LST), from the MYD11A2 product **(C)**; precipitation, from the CHIRPS dataset **(D)**; and friction to human movement, modeled by Weiss *et al.* under assumptions of both access to motorized ground transport **(E)** and no access to such transport **(F)**. Study site locations are shown for context. These rasters were visualized with the `landscapetools` R package.

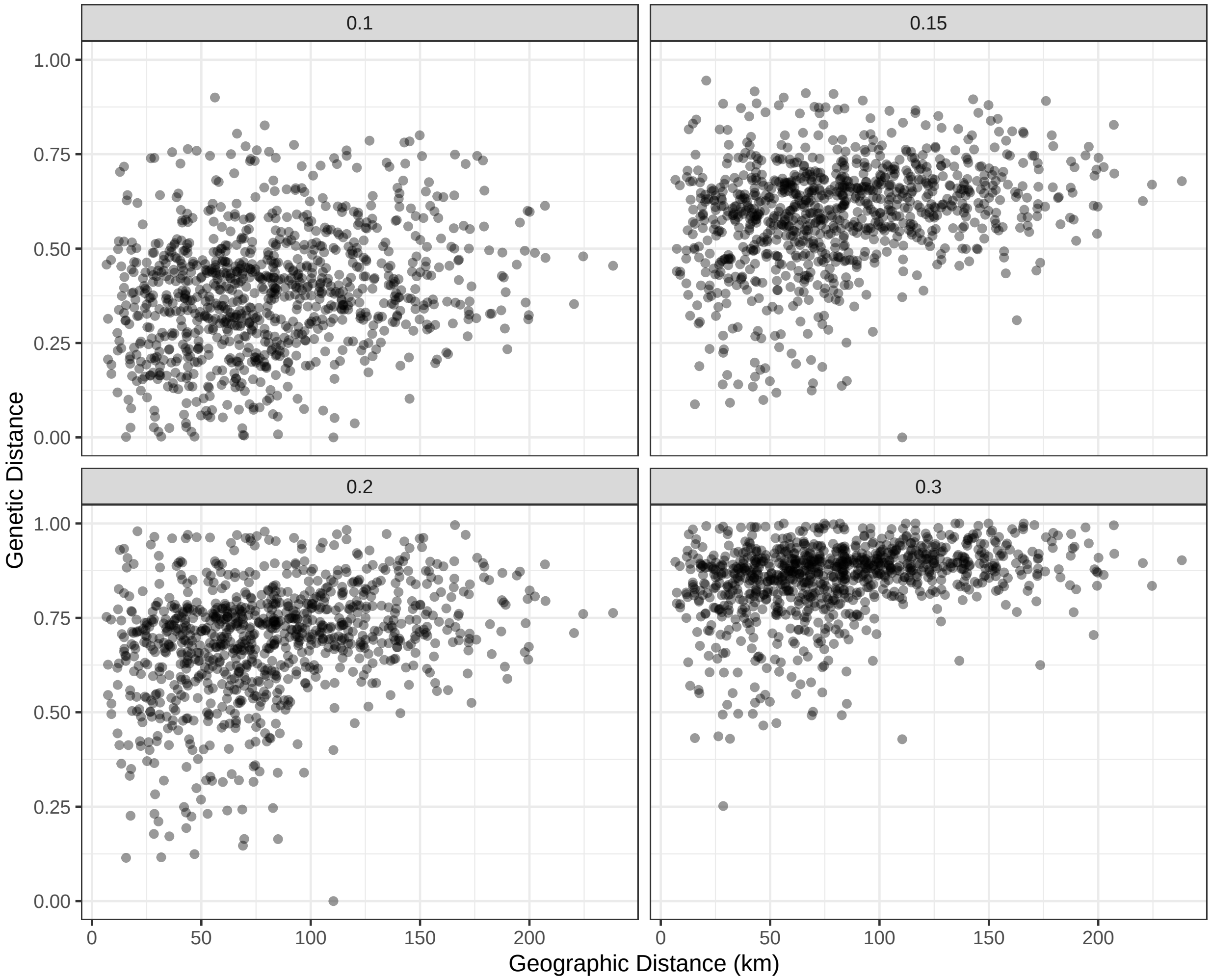

Supplemental Figure 2: Scatterplots of geographic distance versus genetic distance, faceted by the threshold used to define highly-related sample pairs. Points are translucent to aid interpretation. In order to preserve the most information, it is desirable to avoid clustering at either the lower or upper limit of genetic distance. The plot for 0.3 exhibits this type of pattern most clearly, but it is also present in the plots for thresholds of 0.1 and 0.2. Therefore, 0.15 was selected as the best threshold. This plot shows the version of the data corresponding to a minimum of 5 samples per study site, but the version created with a minimum of 15 samples per study site shows a similar pattern.

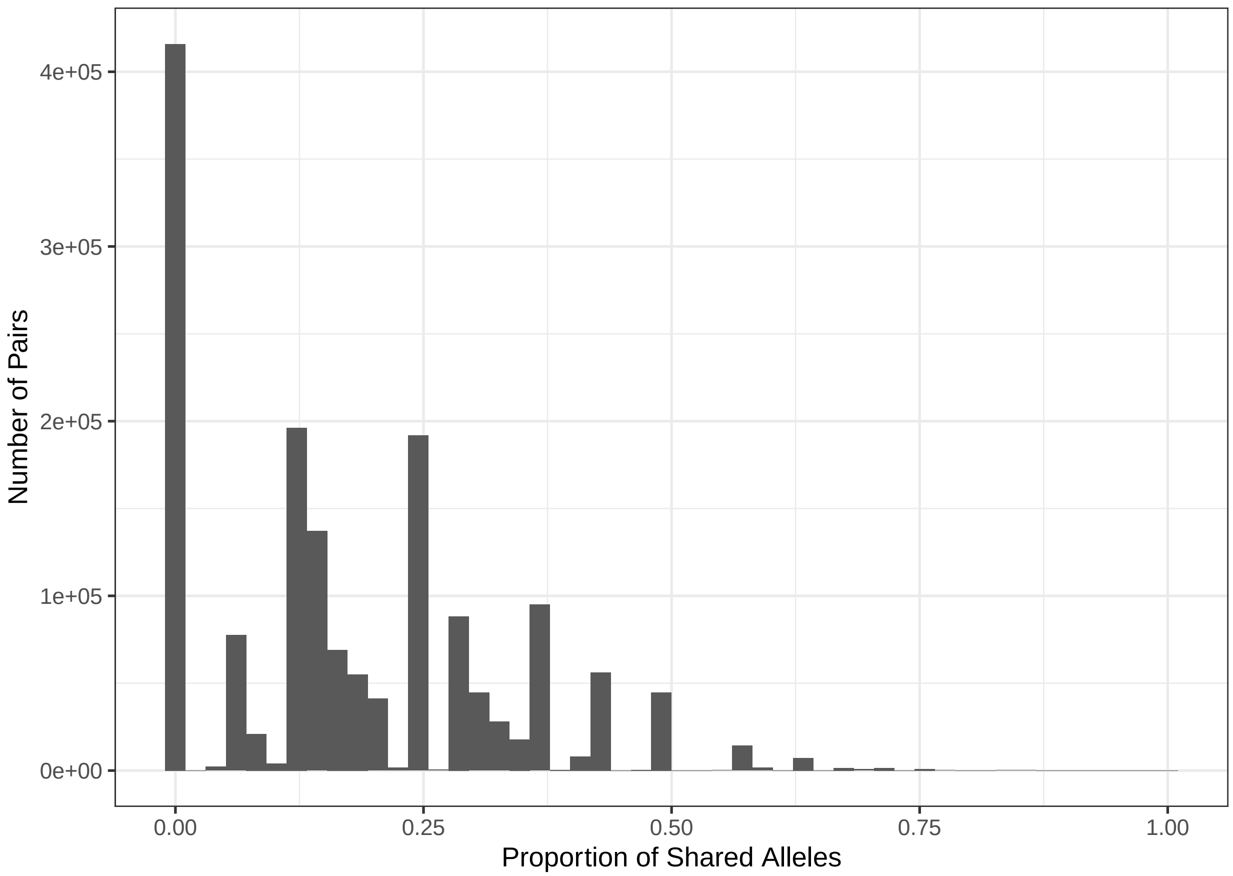

Supplemental Figure 3: Histogram of the proportion of alleles shared between each pair of individuals.

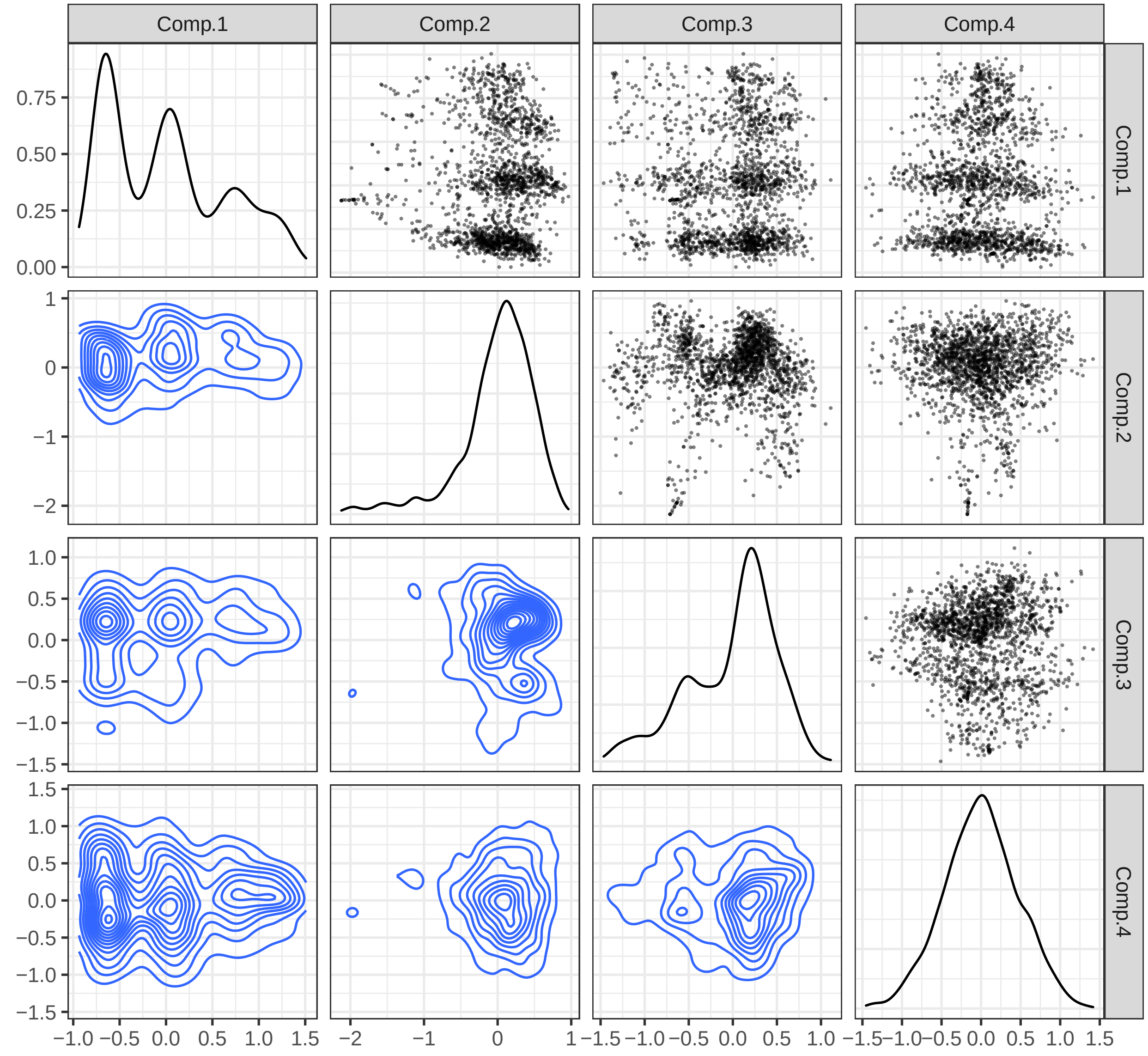

Supplemental Figure 4: This figure depicts the first four components identified with PCA of the allele frequencies. The upper half of this grid contains simple scatter plots of the major components, while the lower half contains contour plots of the same information. Note that the axes for each plot are a unique combination of the two components in question, such that the scatter plot and contour plot for a given component pair visualize the same data but at a 90° angle to one another. Density plots of each component are along the diagonal. This figure was made with the help of the `GGally` R package.

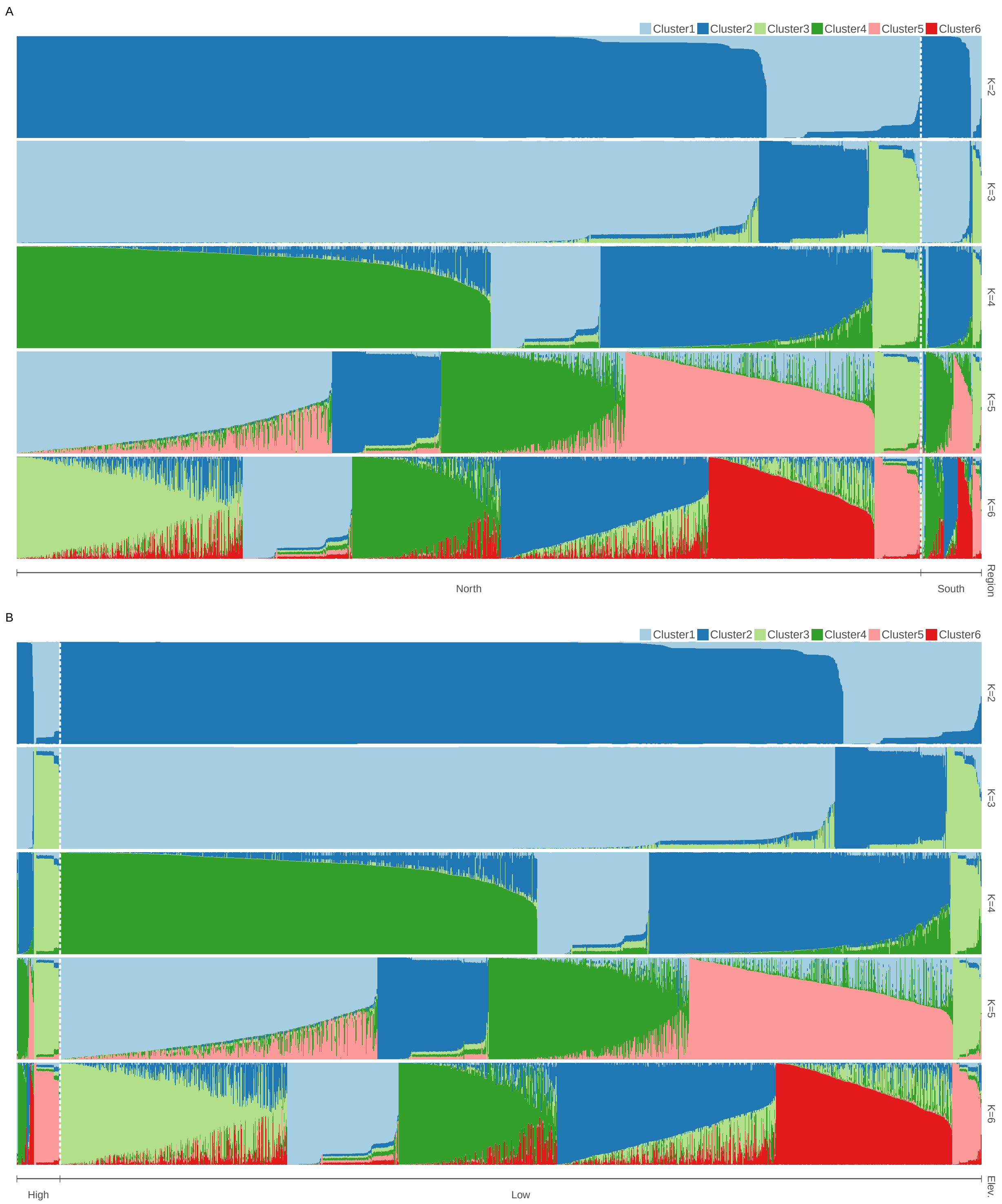

Supplemental Figure 5: Bar plots showing the admixture coefficients estimated by *rmaverick* for each sample. Bars are sorted according to cluster values and two different groupings: geographic portion of the study area **(A)** and elevation **(B)**. "North" and "South" regions were defined relative to the Winam Gulf. "High" elevation was defined as greater than 1750 meters above sea level, whereas samples from sites below this cutoff were labeled as "Low." The 1750 m threshold was selected based on inspection of the distribution of elevations present in the data, with the intention of finding a natural break point between high and low

elevation sites. These plots were created with the `pophelper` R package.

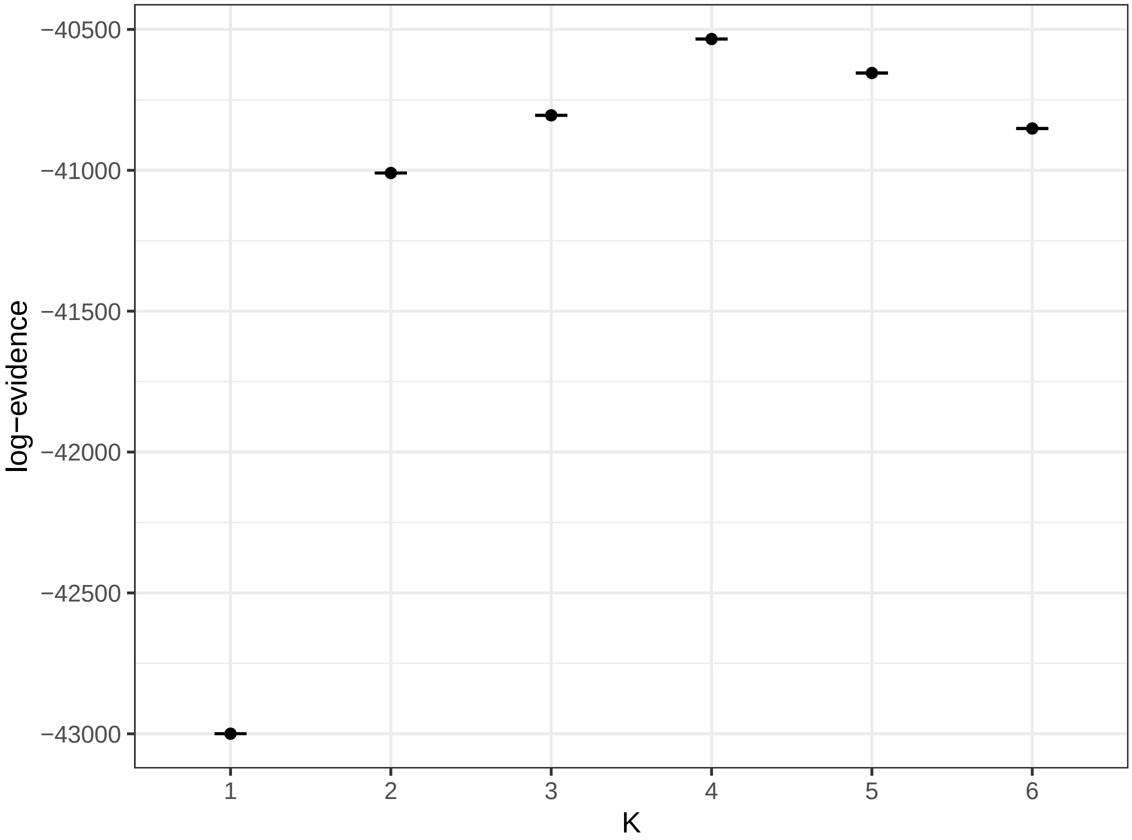

Supplemental Figure 6: Plot of log evidence of the model for each value of K, as estimated by *rmaverick*. Intervals of 95% credibility are indicated by error bars for each K value.
