# Supplementary material for: Implementing landscape genetics in molecular epidemiology to determine drivers of vector-borne disease: A malaria case study
Source: Mol Ecol. Author manuscript; Available in PMC 2023 Dec 4. (PMC10694861; doi:10.1111/mec.16846)
Supplement: Supplemental Text 1:Discriminant Analysis of Principal Components (DAPC) [file NIHMS1941384-supplement-Supplemental_Text_1_Discriminant_Analysis_of_Principal_Components__DAPC_.pdf]

Implementing landscape genetics in molecular epidemiology to determine drivers of vector-borne disease: A malaria case study

Supplemental Text 1: DAPC

Alfred Hubbard Elizabeth Hemming-Schroeder Maxwell Machani
Yaw Afrane Guiyun Yan Eugenia Lo Daniel Janies

Discriminant Analysis of Principal Components (DAPC)

DAPC was performed with the `adegenet` R package (Jombart 2008). This analysis is similar to a PCA, except the components are optimized to emphasize the separation between a specified set of groups. Thus, DAPC finds the pattern of clustering that maximizes the differentiation between the *a priori* groups of samples.

In our case, we used sample sites as our group labels. Before analyzing the DAPC results themselves, two rounds of cross-validation were performed to select the optimal number of principal components. The first was meant to cover a broad range of values, and suggested that the optimal number was around 60. We then performed a second round of cross-validation for every possible number of components between 50 and 70. From this, we determined that 68 components was best, both in terms of successfully predicting group membership and minimizing error.

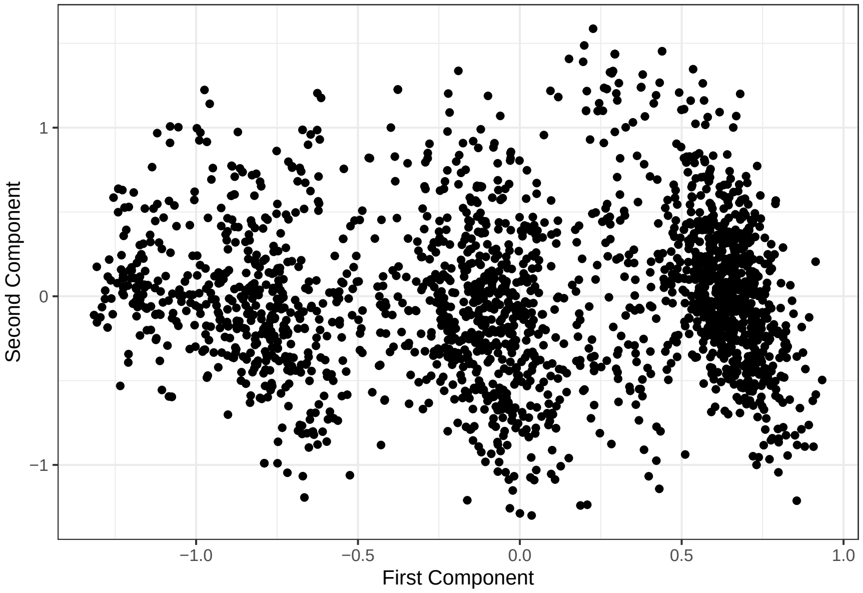

Based on this scatterplot of the first two principal components of the DAPC, there are clearly at least three clusters, with the suggestion of a possible fourth in the upper right. Note that we have not included the usual node labeling seen in DAPC plots - with 44 populations, this is far too cluttered.

References

Jombart, Thibaut. 2008. "Adegenet: A R Package for the Multivariate Analysis of Genetic Markers." *Bioinformatics (Oxford, England)* 24 (11): 1403–5. https://doi.org/10.1093/bioinformatics/btn129.
