# Supplementary material for: Implementing landscape genetics in molecular epidemiology to determine drivers of vector-borne disease: A malaria case study
Source: Mol Ecol. Author manuscript; Available in PMC 2023 Dec 4. (PMC10694861; doi:10.1111/mec.16846)
Supplement: Supplemental Text 2: STRUCTURE [file NIHMS1941384-supplement-Supplemental_Text_2__STRUCTURE.pdf]

Implementing landscape genetics in molecular epidemiology to determine drivers of vector-borne disease: A malaria case study

Supplemental Text 2: STRUCTURE

Alfred Hubbard   Elizabeth Hemming-Schroeder   Maxwell Machani
Yaw Afrane   Guiyun Yan   Eugenia Lo   Daniel Janies

Methods

To provide a reference point for readers of the paper not familiar with *rmaverick*, we present analogous results from the popular Bayesian clustering program STRUCTURE (Pritchard, Stephens, and Donnelly 2000). The objective of this analysis is to identify the set of admixture coefficients for each individual sample that best explain deviations from Hardy-Weinberg and/or linkage equilibrium. These admixture coefficients represent the level of membership in each of K populations. K must be defined in advance, and it is common practice to test several values of K. We ran STRUCTURE for values of K from one to six, based on our ordination analyses and to be consistent with *rmaverick*.

It is also important to run the model for long enough for parameter values to stabilize, both during the “main” model run and during a preliminary burn-in phase that serves to prevent the initial conditions from biasing the results. Based on the stability of model parameters at the end of both the burn-in and sampling phases, we selected a burn-in period of 500,000 iterations and a sampling period of 725,000 iterations.

Mono- and biclonal infections were incorporated by running STRUCTURE with a ploidy of two and repeating the allele when only one was present at a given loci. Samples with more than two clones were discarded for this analysis.

STRUCTURE provides various population models. We used the standard admixture model, the LOCPRIOR model that includes sample location (Hubisz et al. 2009), and the correlated allele frequencies model (Falush, Stephens, and Pritchard 2003).

With these settings, we ran STRUCTURE 10 times for each K value. The highest likelihood run for each K was selected for further analysis.

Results

Bar Plots

With the aid of the `pophelper` R package (Francis 2016), we first visualized our results using the bar plots that are standard in STRUCTURE analyses.

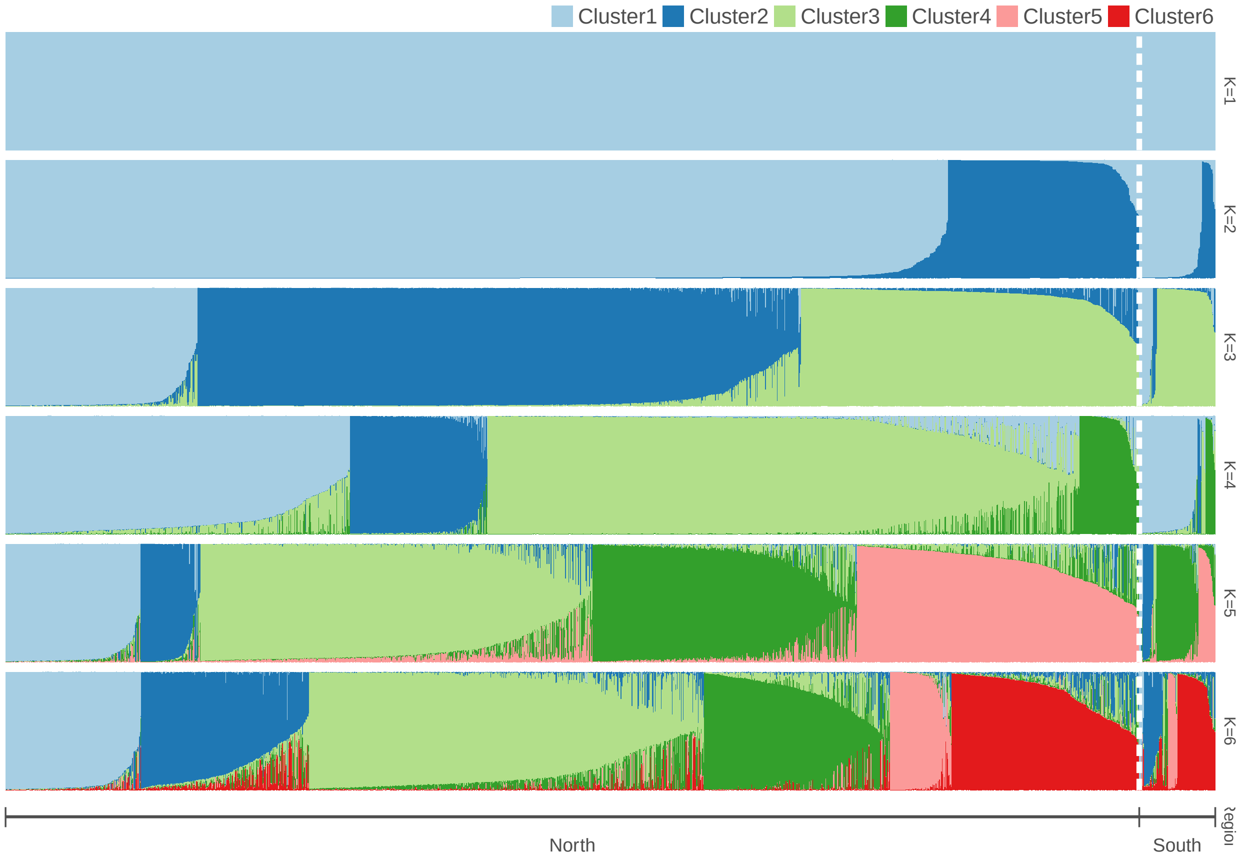

The bars in these plots show the admixture coefficients for each individual. They have been sorted according to cluster values and whether the sample was collected north or south of Lake Victoria. One bar plot is shown for each K value. Similar to the results from *rmaverick*, these plots do reveal different patterns of population membership north and south of the lake.

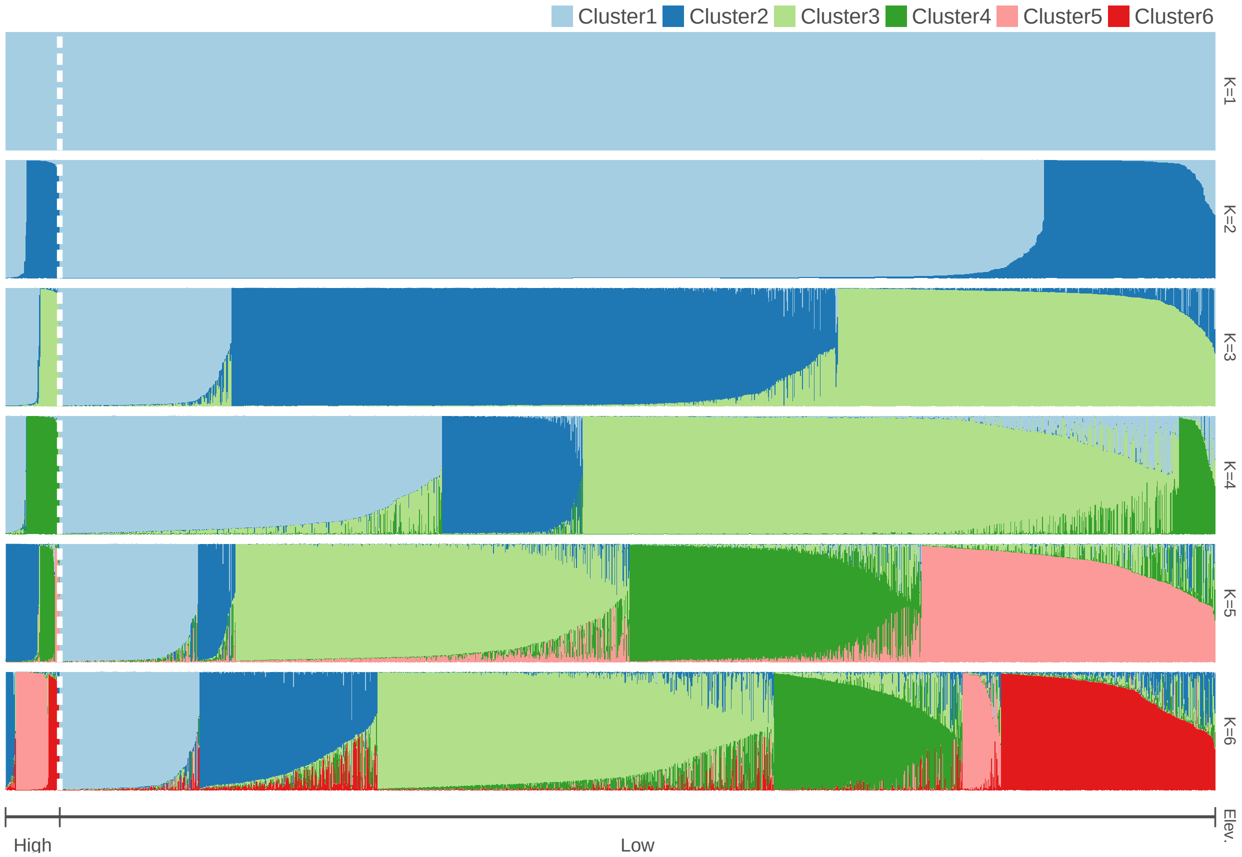

These bar plots show the same data as above, except they are grouped according to elevation, with samples collected above 1750 m defined as "High" and samples collected below that elevation defined as "Low." Similar to the plot grouped according to region, some of the clusters are evidently more or less common depending on elevation.

Pie Chart Map

To show the geographic patterns hinted at above more clearly, we created a map with pie charts at each study site showing the average admixture coefficents for samples collected from that site. This is analogous to Figure 1 in the main paper. We created this map for $K=4$ to be consistent with our analysis of the *rmaverick* results. This map was made with the help of the `scatterpie` R package (Yu 2021). Country boundaries, included for context, were obtained with the `rnaturalearth` R package (South 2017). The background is Esri's world shaded relief layer (© 2009 ESRI).

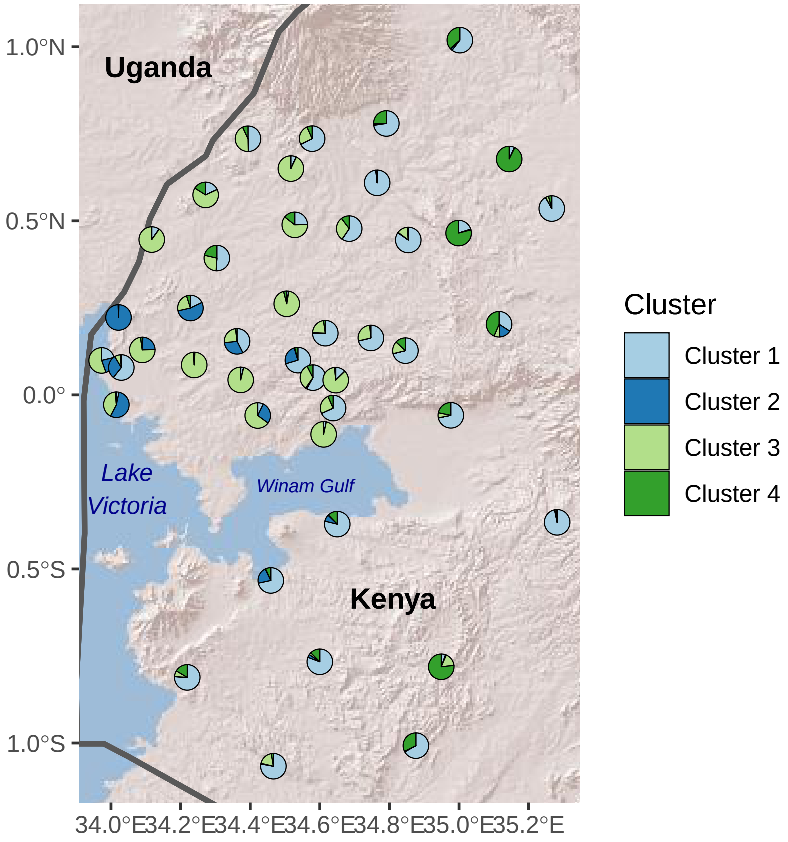

The patterns shown by this map are quite similar to those in Figure 1 from the main paper. One cluster, Cluster 2, is disproportionately associated with samples collected from the west, near the border with Uganda. Cluster 3 is primarily present in the lowlands north of the Winam Gulf, and overlaps considerably with Cluster 2. Cluster 4 is mostly found in the high elevation sites in the eastern part of the study area. Cluster 1 is the only group that does not show a strong geographic pattern.

References

Falush, Daniel, Matthew Stephens, and Jonathan K. Pritchard. 2003. "Inference of Population Structure Using Multilocus Genotype Data: Linked Loci and Correlated Allele Frequencies." *Genetics* 164 (4): 1567–87. https://doi.org/10.1093/genetics/164.4.1567.

Francis, R. M. 2016. "Pophelper: An R Package and Web App to Analyse and Visualize Population Structure." *Molecular Ecology Resources* 17 (1): 27–32. https://doi.org/10.1111/1755-0998.12509.

Hubisz, Melissa J., Daniel Falush, Matthew Stephens, and Jonathan K. Pritchard. 2009. "Inferring Weak Population Structure with the Assistance of Sample Group Information." *Molecular Ecology Resources* 9 (5): 1322–32. https://doi.org/10.1111/j.1755-0998.2009.02591.x.

Pritchard, J. K., M. Stephens, and P. Donnelly. 2000. "Inference of Population Structure Using Multilocus Genotype Data." *Genetics* 155 (2): 945–59. https://doi.org/10.1093/genetics/155.2.945.

South, Andy. 2017. *Rnaturalearth: World Map Data from Natural Earth.* https://CRAN.R-project.org/package=rnaturalearth.

Yu, Guangchuang. 2021. *Scatterpie: Scatter Pie Plot.* https://CRAN.R-project.org/package=scatterpie.
